# *Dicyema* Pax6 and Zic: tool-kit genes in a highly simplified bilaterian

**DOI:** 10.1186/1471-2148-7-201

**Published:** 2007-10-25

**Authors:** Jun Aruga, Yuri S Odaka, Akiko Kamiya, Hidetaka Furuya

**Affiliations:** 1 Laboratory for Comparative Neurogenesis, RIKEN Brain Science Institute, Wako 351-0198, Japan; 2 Department of Biology, Graduate School of Science, Osaka University, Toyonaka, Osaka 560-0043, Japan

## Abstract

**Background:**

Dicyemid mesozoans (Phylum Dicyemida) are simple (8–40-cell) cephalopod endoparasites. They have neither body cavities nor differentiated organs, such as nervous and gastrointestinal systems. Whether dicyemids are intermediate between Protozoa and Metazoa (as represented by their "Mesozoa" classification) or degenerate species of more complex metazoans is controversial. Recent molecular phylogenetic studies suggested that they are simplified bilaterians belonging to the Lophotrochozoa. We cloned two genes developmentally critical in bilaterian animals (Pax6 and Zic), together with housekeeping genes (actin, fructose-bisphosphate aldolase, and ATP synthase beta subunit) from a dicyemid to reveal whether their molecular phylogeny supported the "simplification" hypothesis, and to clarify evolutionary changes in dicyemid gene structure and expression profiles.

**Results:**

Genomic/cDNA sequence analysis showed that 1) the Pax6 molecular phylogeny and Zic intron positions supported the idea of dicyemids as reduced bilaterians; 2) the aa sequences deduced from the five genes were highly divergent; and 3) *Dicyema *genes contained very short introns of uniform length. In situ hybridization analyses revealed that *Zic *genes were expressed in hermaphroditic gonads, and *Pax6 *was expressed weakly throughout the developmental stages of the 2 types of embryo and in the hermaphroditic gonads.

**Conclusion:**

The accelerated evolutionary rates and very short and uniform intron may represent a part of *Dicyema *genomic features. The presence and expression of the two tool-kit genes (*Pax6 *and *Zic*) in *Dicyema *suggests that they can be very versatile genes even required for the highly reduced bilaterian like *Dicyema*. Dicyemids may be useful models of evolutionary body plan simplification.

## Background

**Figure 1:**
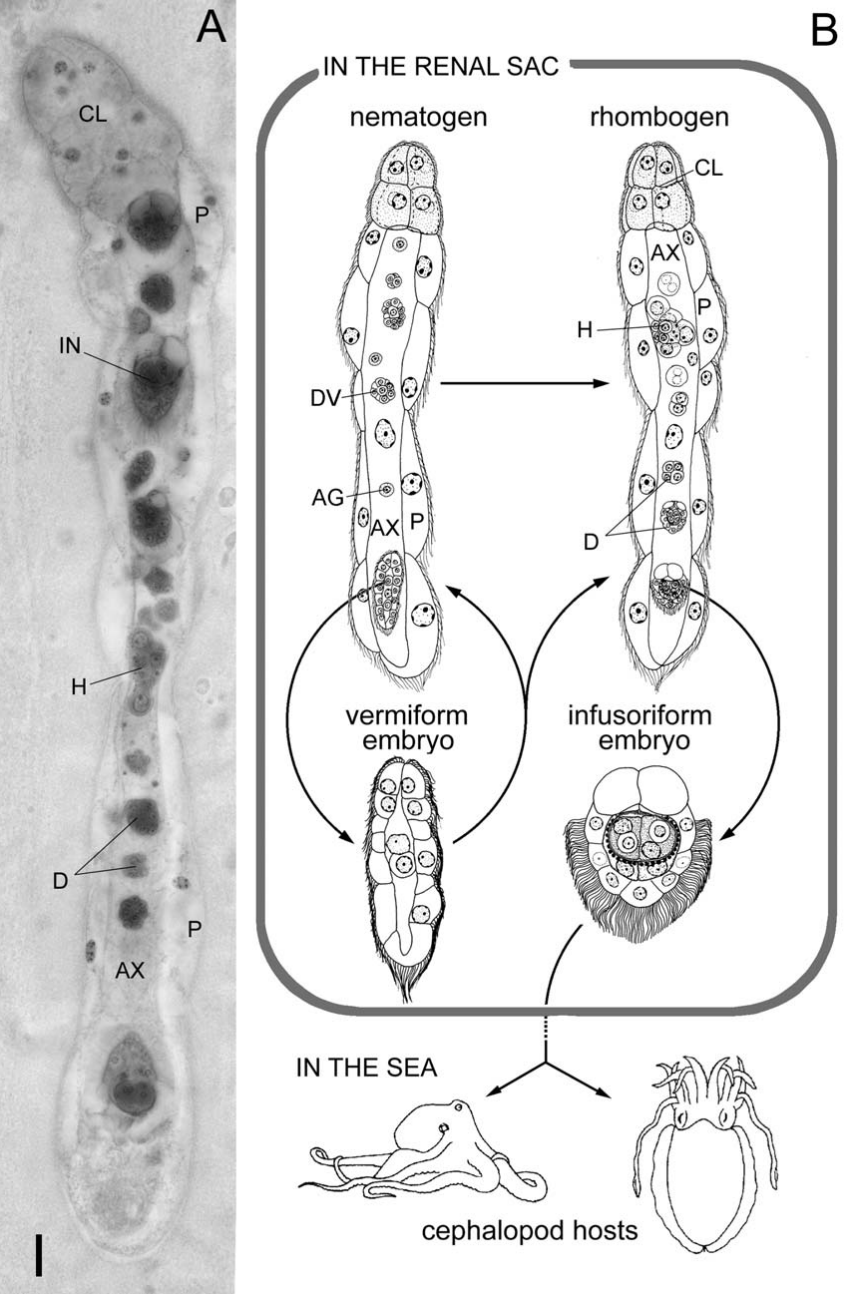
***Dicyema acuticephalum***. (A) Light micrograph of a rhombogen. Note that embryos develop in the axial cell. Scale bar, 10 μm. (B) Life cycle of the dicyemid (after Furuya and Tsuneki [1]). The dotted line indicates an unknown process. The vermiform stage includes the nematogen, rhombogen, and vermiform embryo. The development of infusorigens, gametogenesis around the infusorigen, and development of the 2 types of embryo all proceed within the axial cell.*AG*, agamete; *AX*, axial cell; *CL*, calotte; *D*, developing infusoriform embryo; *DV*, developing vermiform embryo;*H*, hermaphroditic gonad (infusorigen); *IN*, infusoriform embryo; *P*, peripheral cell.

Dicyemid mesozoans (Phylum Dicyemida) are typically found in the kidneys of cephalopod mollusks [For general reviews on Dicyemid, [1]]. They have neither body cavities nor differentiated organs, such as nervous and gastrointestinal systems (Fig. 1A). Their life cycle consists of two phases (Fig. 1B). One is the vermiform stage, in which the dicyemid exists as a vermiform embryo formed asexually from an agamete to form worms in the renal sac of the host. The other is the infusoriform larva, which develops from a fertilized egg produced around hermaphroditic gonads called infusorigens and can escape from the host into seawater. How the infusoriform larvae enter the vermiform stage in the new host is unknown. However, high population density in the cephalopod phase may cause the shift from an asexual mode to a sexual mode of reproduction. Notably, both fertilization and embryonic development occur within the worm body. The viviparous mode of reproduction makes this organism a good subject for developmental analysis [2].

The original classification of dicyemids as Mesozoa reflects their intermediate position between the Protozoa (unicellular eukaryotic organisms) and Metazoa (multicellular animals) in body organization. The phylogeny of the dicyemids is controversial, and some researchers consider that dicyemids represent truly primitive multicellular organisms. However, several zoologists regard the simple body plan of dicyemids as the result of specialization of parasitism [references in [1]]. Recent molecular studies suggest that dicyemids are not truly primitive animals. 18s rDNA phylogenetic analyses by Katayama et al. [3] showed that the dicyemids belong among the bilateria, and from the structure of the amino acid (aa) sequence in the carboxy-terminal flanking region of the Antennapedia protein in *Dicyema orientale*, Kobayashi et al. [4] suggested their affinity to the Lophotrochozoa. A limited number of genes are available for phylogenetic analysis, and the phylogenetic relationships of the dicyemids will need further evaluation when the sequences of more genes become available.

If reduction of body plan complexity secondary to parasitism truly happened in dicyemids, we expect to find genomic features in the dicyemid genome that are associated with the adaptive simplification of body organization. However, such features have not been described in dicyemids. In broader terms, the genomic basis of the simplification that occurs during the course of evolution is poorly understood.

To determine the genomic changes that may occur during the simplification of body organization, we focused on *Pax6 *and *Zic *(both of which encode proteins that regulate developmentally critical processes in diverse bilaterians) in dicyemids. Pax6 plays key roles in the development of sensory organs and the nervous system [reviewed in [5]]. In particular, *Pax6 *is a master gene in eye development in both vertebrates and *Drosophila*. In contrast, Zic family proteins have diverse roles [reviewed in [6,7]]. In vertebrates, they are crucial for embryonic patterning as well as neural tube formation, neural crest generation, and mesodermal segmentation. In urochordates, Zic proteins are required for cell fate decisions for differentiation into various cell lineages, including nerve, muscle, and notochord tissues. In protostomes, *Zic *genes are essential for embryonic segmentation, midgut morphogenesis, adult head formation in *Drosophila *[8-10], and vulva formation in *Caenorhabditis *[11]. Both *Pax6 *and *Zic *have structurally related genes in cnidarians; the possible involvement of these cnidarian genes in developmental processes analogous to those controlled by their triploblast homologs has been proposed [12,13].

Here, we investigated the structures of the dicyemid *Pax6 *and *Zic *genes and 3 housekeeping genes [*actin*, *ATP synthase beta subunit *(*ATPS*), and *fructose-bisphosphate aldolase *(*aldolase*)], together with the Pax6 and Zic expression patterns. Our results should add to knowledge of the phylogenetic position, common genomic features of the dicyemids, and the role of *Pax6 *and *Zic *in their ontogeny.

## Results

### Amino acid sequences of Dicyema Pax6 and Zic proteins

**Figure 2:**
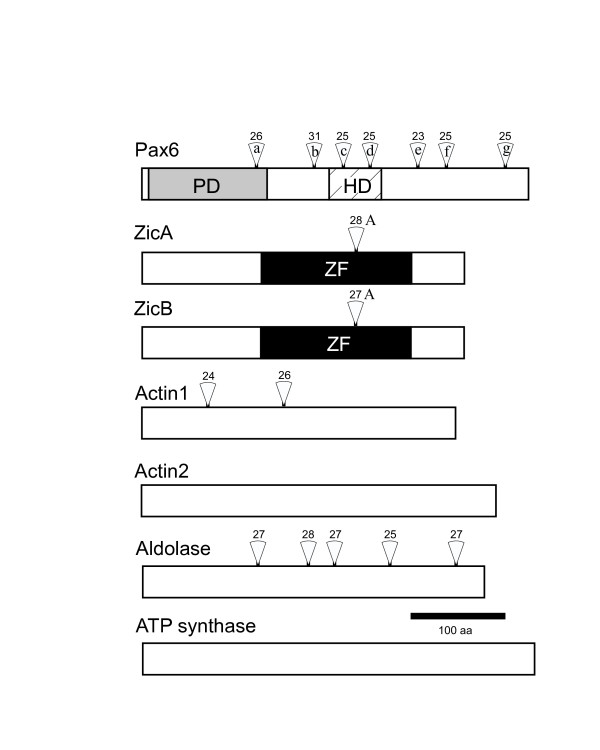
**Dicyemid genes cloned in this study**. The entire protein-encoding (*open box*) domains and the functional domains (ZF, zinc finger domain, *black*; PD, paired box, *gray*; HD, homeodomain, *hatched*) are drawn proportionally. Positions of introns are indicated by open triangles. The number above each open triangle indicates the size (in nucleotides) of the intron at that position. *actin2 *and *ATP synthase *lack introns. Scale bar, 100 aa.

We cloned *Dicyema *orthologs of the *Pax6*, *Zic*, and *actin *genes by PCR amplification of *Dicyema acuticephalum *cDNA and genomic DNA. Both genomic and cDNA fragments containing entire open reading frames were cloned and sequenced to reveal the entire aa sequences encoded and the exon-intron boundaries (Fig. 2). Two types of *Zic *and *actin *genes were identified; they were termed *ZicA*/*ZicB *and *actin1*/*actin2*, respectively. In the course of the cloning procedure, we fortunately obtained sequences representing 2 more genes, the housekeeping genes *ATPS *and *aldolase*, both of which were fully cloned to supplement the analysis (Fig. 2).

**Figure 3:**
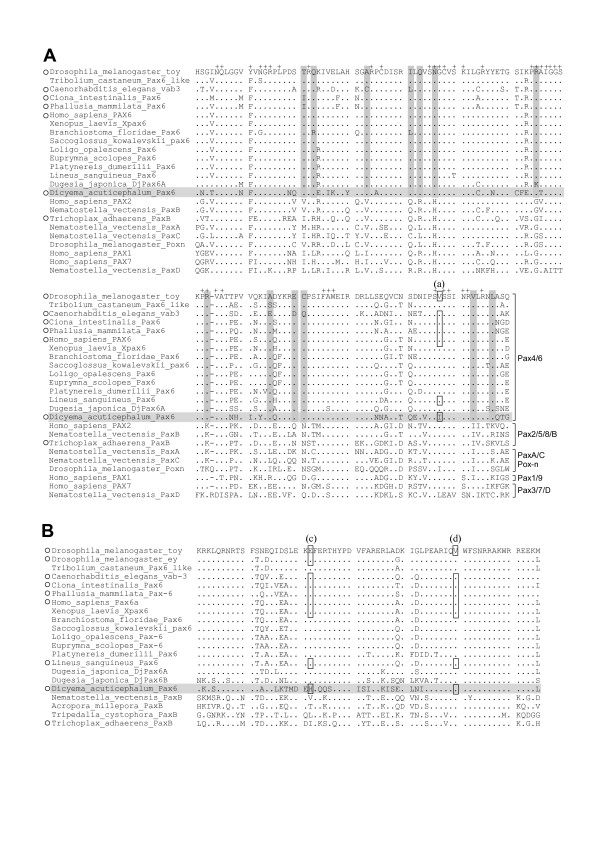
**Multiple alignment of Pax6 aa sequences**. The sequences corresponding to Pax6 PD (A) and HD (B) were aligned. *Dots *indicate aa residues identical to those of the top line (*Drosophila*_toy).*Dashes *indicate artificial spaces inserted by the alignment program. *+ *indicates the residue that interact with DNA [14]. *Horizontal shaded lines *indicate the *Dicyema *sequence. *Vertical shaded lines *indicate aa residues strongly conserved in Pax6 subfamily, but not in the others.*Open boxes *represent the positions of introns. The phases of introns are (a), 0; (c), 1; (d), 0. *Open circles *indicate animals in which intron positions were examined.

We first examined the conserved domains of the putative aa sequence of *Dicyema *Pax6 (408 aa), which contained two conserved domains, the paired box domain (PD, cd00131.3) and homeodomain (HD, cd00086.3), as do Pax6 proteins from other animals. Both domains act as DNA-binding domains. Alignment of the putative aa sequences of the *Dicyema *Pax6 PD (Fig. 3A) and HD (Fig. 3B) with those of known metazoan Pax sequences revealed marked similarity among these domains. DNA-interacting aa residues have been determined in human PAX6 PD by a crystal structure analysis [14]. Conservation between the human and *Dicyema *sequences was more strongly observed in the DNA-contacting aa residues (36/37) than in non-contacting residues (64/92) (*P *< 0.001 by Fisher's exact test), suggesting the functional importance of the former type of residues. aa sequences other than those of PD and HD showed no clear similarity to those encoded by other *Pax6 *orthologs (data not shown).

**Figure 4:**
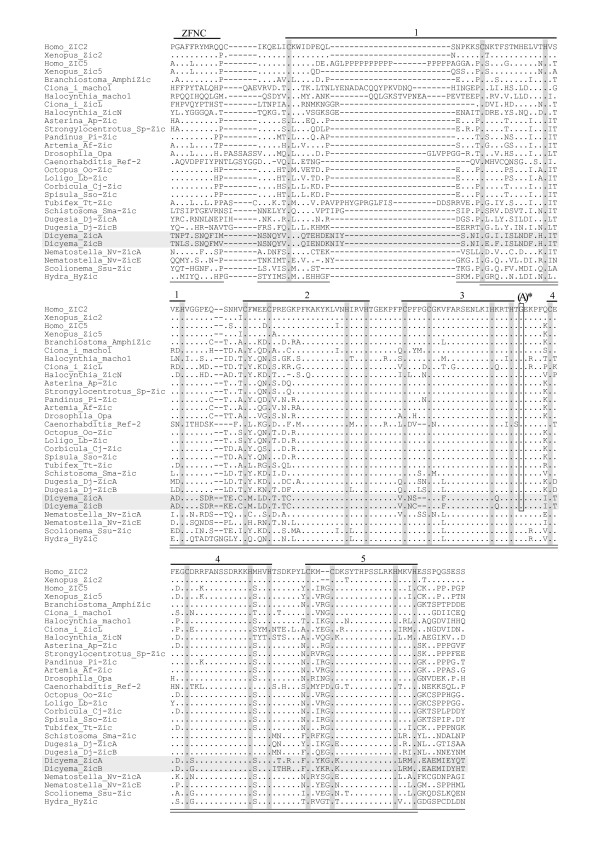
**Multiple alignment of aa sequences corresponding to Zic ZF and flanking regions**. *Dots *represent aa residues identical to those in the top lines (*Homo*_ZIC2). *Dashes *indicate artificial spaces inserted by the alignment program. *Horizontal shaded lines *indicate the *Dicyema *Zic sequences. *Vertical shaded lines *indicate the positions of cysteine and histidine residues in the C2H2 motif. Labels above the top lines indicate subregions (*ZFNC*, Zinc finger N-terminal conserved region; *1–5*, C2H2 ZF motif 1–5). Open box labeled with (A)* indicates the position of the bilaterian-specific intron (A-intron) [15], where the exon-intron boundaries for the *Halocynthia*, *Octopus*, and *Dugesia *sequences have not yet been clarified. Cnidarian *Zic *genes (*Nematostella*, *Scolionema*, and *Hydra*) lack the intron in this position. *Bottom parallel lines *indicate the region subjected to the aa substitution rate analysis. *Bottom underlines *and *parallel lines *indicate the region used for the NJ tree analysis.

*Dicyema *ZicA (340 aa) and ZicB (340 aa) showed strong similarity to each other (87% aa matching over the entire proteins). A homology search analysis with Zic family proteins in other species revealed that the region of greatest similarity was confined to a zinc finger (ZF) domain comprising tandem repetitions of 5 C2H2 ZF motifs (ZF1–5) (Fig. 4 and data not shown); other regions, including the Zic-Opa-Conserved (ZOC) and ZF-N-terminal flanking-Conserved (ZFNC) domains, lacked noteworthy similarity with other Zic family members [15]. In the multiple alignment of ZF and ZFNC from various species (Fig. 4), *Dicyema *ZicA and ZicB showed clear similarity to other Zic proteins in the ZF2–5 region, which is strongly conserved among all Zic proteins identified so far. The number of aa residues between the two cysteines of the ZF1 C2H2 motif was 15. This structure is analogous to those of other Zic proteins that have highly variable numbers in this region [ranging from 6 to 38 in15] and is different from those of Gli/Glis proteins (4 in the corresponding region), which share similar ZF domain sequences with Zic family proteins.

We identified two *Dicyema actin *genes that encode actin1 (332 aa) and actin2 (376 aa) proteins (data not shown). The difference in sequence length reflects the presence or absence of an N terminal region, and the homology in the overlapping region was 89% at the aa level.

### Exon-intron organization of the *Dicyema *genes

In total, there were 16 spliceosomal introns in the protein coding regions of the 7 genes (Fig. 2). The size of the introns was a surprising feature, as they were generally short and distributed over a narrow range (mean length, 26.2 ± 1.9 nucleotides, n = 16; Fig. 2). This intron length may be representative of the major population of intron sizes in dicyemids.

The positions of introns in the conserved domains of *ZicA*, *ZicB*, and *Pax6 *were identical to those previously identified as evolutionarily conserved intron positions in each gene family [15-18]. The *Dicyema ZicA *and *ZicB *genes both possessed a single intron in the center of the evolutionarily conserved ZF domain (Figs. 2, 4). The positions of these introns corresponded to that of the A-intron, which was conserved in all 32 *Zic *genes from 7 different bilaterian phyla but was not found in the 8 cnidarian *Zic *genes evaluated [15]. *Dicyema Pax6 *had 7 introns (a-g) in the putative protein-coding region (Figs. 2 and 3). Of the 7 introns, one (a) was located in PD and 2 (c, d) were in HD. The positions of these 3 introns matched those of the introns of evolutionarily conserved *Pax *genes from a broad range of eumetazoans [17-19].

### Phylogenetic analysis utilizing the *Dicyema *aa sequences

We next tested whether the *Dicyema *sequences obtained are useful to understand the phylogenetic position of *Dicyema*. All of the deduced aa sequences were subjected to molecular phylogenetic analyses based on the neighbour-joining (NJ), Bayesian inference (BI), and maximum parsimony (MP) methods. A support for the bilaterian origin was obtained in the case of the Pax6 molecular phylogeny.

**Figure 5:**
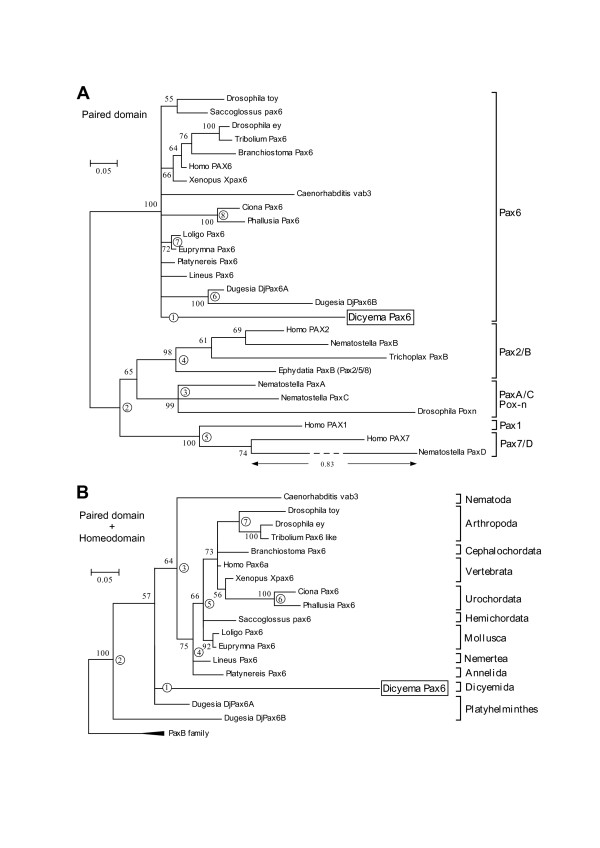
**BI tree based on metazoan Pax6-related aa sequences**. BI trees of Pax6 were drawn by MrBayes [44] by using PD sequences (A) and concatenated PD+HD sequences (PD+HD). PD tree was unrooted while PD+HD tree was rooted with PaxB family of which subtree is indicated in closed triangle. *Numbers next to interior branches *indicate posterior probability. *Circled numbers *indicate the Dicyema-branch-regrafted position in the alternative tree analyses (Tables 1 and 2). *Scale bar *represents evolutionary distance in substitutions/aa residue. Classification of Pax genes according to a previous study [20] is indicated in the right side of (A).

The metazoan Pax family sequences can be classified into five groups [20]. For the molecular phylogenetic analysis of *Dicyema *Pax6, we first used 129–130 aa PD sequences. *Dicyema *Pax6 sequences, previously known Pax6 sequences, and representative Pax sequences from the other groups. The phylogenetic trees by BI (Fig. 5A) and NJ [see Additional file 1] methods, commonly grouped *Dicyema *Pax6 with other Pax6 sequences. We also performed the analysis using the concatenated PD and HD sequences (194 aa) from Pax6 family and its closest relatives, cnidarian and placozoan PaxB sequences (Fig. 5B). *Dicyema *Pax6 was grouped with Pax6 family commonly in the BI (Fig. 5B), NJ [see Additional file 1], and MP [see Additional file 1] trees. Deciphering more detailed phylogenetic position within the bilaterian was not possible in the current analysis.

**Table 1:** Shimodaira-Hasegawa test for Pax6 BI trees in Fig. 5A Paired domain tree

Tree	Differential logL	logL	P value	Significantly worse?
1	-2617.8	-4.6	0.756	No
2	-2657.6	-44.3	0.002	Yes
3	-2671.0	-57.7	0.000	Yes
4	-2666.4	-53.1	0.000	Yes
5	-2661.6	-48.3	0.001	Yes
6	-2617.9	-4.6	0.669	No
7	-2626.7	-13.5	0.328	No
8	-2613.3 <---best			

**Table 2:** Shimodaira-Hasegawa test for Pax6 BI trees in Fig. 5B Paired domain + Homeodomain tree

Tree	Differential logL	logL	P value	Significantly worse?
1	-3004.0 <---best			
2	-3005.3	-1.3	0.696	No
3	-3005.0	-1.0	0.825	No
4	-3014.2	-10.2	0.292	No
5	-3011.8	-7.7	0.404	No
6	-3026.6	-22.6	0.039	Yes
7	-3014.9	-10.8	0.270	No

To confirm the robustness of the trees, we replaced the *Dicyema *branch to other places in the BI tree, and performed the likelihood ratio test developed by Shimodaira and Hasegawa [21,22]. The results indicated that the placement of the *Dicyema *branch to other bilaterian clades did not significantly worsen the tree except placing in urochodate clade whereas its relocation to non-Pax6 clades worsen the likelihood scores (Tables 1 and 2).

**Figure 6:**
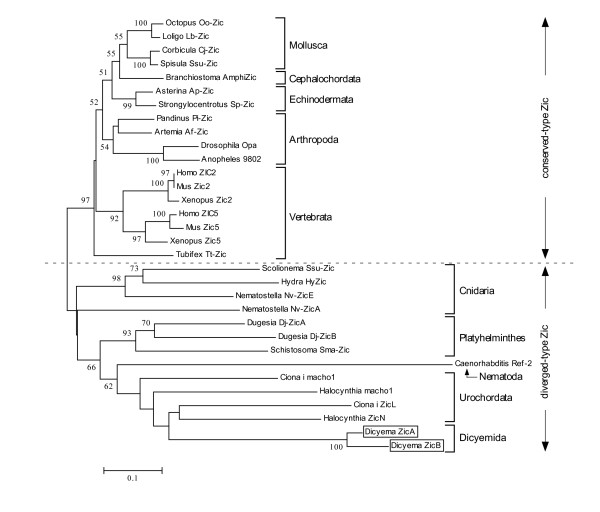
**NJ tree based on metazoan Zic-related aa sequences**. NJ tree of Zic was drawn by MEGA3 [43] by using Zic ZF core sequences (double underlined region in Fig. 4). The evolutionary distances were determined using the JTT matrix [46]. The internal labels indicate the percentage value of bootstrap test (1000 replicates). *Scale bar *represents evolutionary distance in substitutions/aa residue. Classification of Zic genes according to a previous study [15] is indicated by *broken line *and *arrows*.

In comparison to the Pax6, molecular phylogenetic analysis of Zic and the three house keeping proteins did not provide us any insights concerning the phylogenetic position of *Dicyema *(Fig. 6) [see Additional file 1]. In the case of Zic NJ tree (Fig. 6), *Dicyema *was grouped with diverged type Zic protein [15]. However, the phylogenetic relationship of diverged type of Zic proteins are highly mixed up with low statistical support.

### Accelerated evolutionary rate in *Dicyema *species

**Figure 7:**
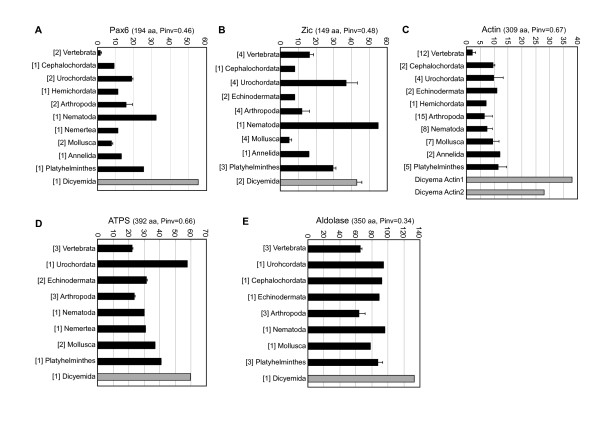
**Amino acid substitution rates in relation to bilaterian ancestor sequences**. In each graph, the x-axes indicate mean numbers of substituted aa in comparison to ancestor sequences in each taxa, with standard deviations (error bars). A, Pax6 PD+HD; B, Zic ZF; C, actin; D, ATPS; E, Aldolase. *Bracketed numbers *left of the taxa names indicate the numbers of sequences subjected to the analysis. *aa*, length of aa sequences subjected to the analysis; *Pinv*, proportion of invariant aa among all tested sequences. The animal species are listed with their sequence accession numbers [see Additional file 2].

While conducting the molecular phylogenetic analysis, we noticed that the *Dicyema *genes were always placed with long branches in the phylogenetic trees. To test whether the evolutionary rate in *Dicyema *was higher than those of the other groups of animals, we compared the number of substituted aa residues with the number of bilaterian ancestor aa sequences predicted by the ANCESCON program [23]. For this purpose, the available sequences from major bilaterian phyla were collected from the NCBI databases, excluding paralogs to uniformly represent phylogenetic variances. The comparative analysis included the 7 sequences encoded by the 5 gene groups (Fig. 7). Among the bilaterian orthologs, the aa sequence of the 3 housekeeping genes (*actin*, *ATPS*, and *aldolase*) from *Dicyema *generally showed the highest degree of sequence divergence from the putative bilaterian ancestor sequences (Fig. 7C,D,E). The differences in the aa substitution frequencies between the *Dicyema *and the other bilaterian sequences were statistically significant (actin, *P *< 1 × 10^-37^; ATPS, *P *< 1 × 10^-7^; aldolase, *P *< 1 × 10^-10^, by chi-squared tests). The divergence rates of the *Dicyema *actin sequences were particularly high [5.3 or 3.9 times (actin1 and actin2, respectively) higher than the means of the other bilaterian sequences], compared with those of ATPS and aldolase (2.1 and 1.7 times higher, respectively).

The *Dicyema *Pax6, ZicA, and ZicB aa sequences had divergence rates 3.6, 2.1, and 2.3 times higher than the means of these sequences in the other bilaterians, supporting the hypothesis that the sequence divergence rate is increased among the proteins encoded by *Dicyema *genes. However, in the case of Zic and Pax6, the divergence rates varied among the bilaterian phyla. The variance in divergence rates over all of the common animal taxa differed strongly between *Zic *and the 3 housekeeping proteins [actin, *P *= 0.0056 (n = 10); ATPS, *P *= 0.057 (n = 8); aldolase, *P *= 0.0126 (n = 9) in F-tests].

### Expression patterns of *ZicA, ZicB, Pax6*, and actin

**Figure 8:**
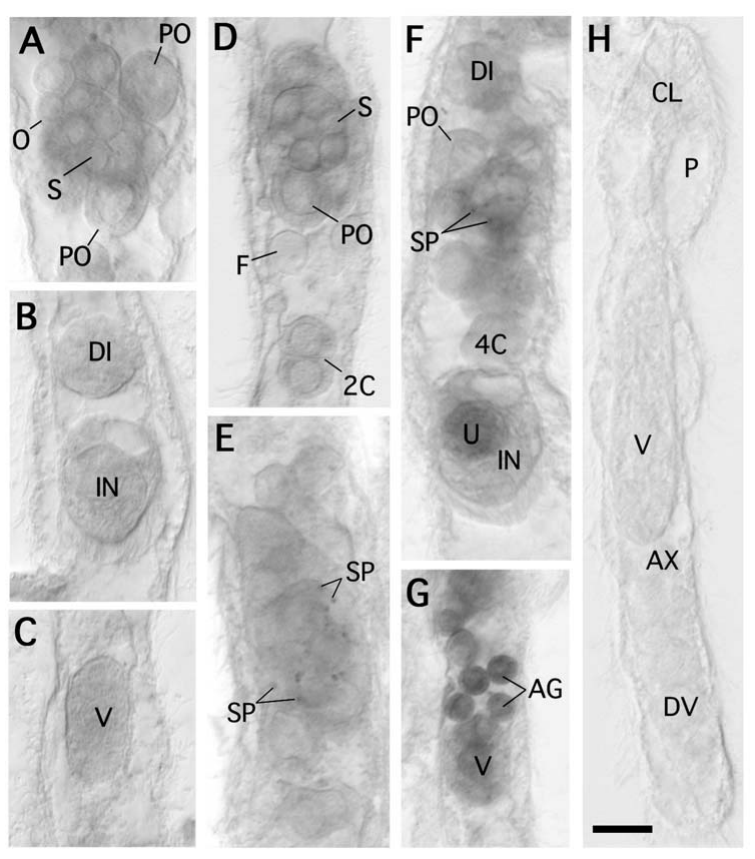
**In situ hybridization of dicyemid ZicA, ZicB, Pax6, and actin1**. (A-C) *ZicA*; (D) *ZicB*; (E) *Pax6*; (F, G) *actin1*; (H) *in situ *hybridization, without RNA probe as a negative control. (A) infusorigen; (B) later stage of infusoriform embryo and formed infusoriform embryo; (C) later stage of vermiform embryo; (D) infusorigen and 2-cell-stage embryo; (E), infusorigen; (F), infusorigen and early stages of developing infusoriform embryos, and formed infusoriform embryo; (G), agametes and later stage of vermiform embryo; (H), whole body of young individual. Scale bar, 10 μm. *AG*, agamete; *AX*, axial cell; *CL*, calotte; *DI*, developing infusoriform embryo; *DV*, developing vermiform embryo; *F*, fertilized egg; *IN*, infusoriform embryo; *O*, oogonium; *PO*, primary oocyte; *P*, peripheral cell; *S*, spermatogonium; *SP*, sperm; *U*, urn cell; *V*, vermiform embryo; *2C*, 2-cell-stage embryo; *4C*, 4-cell-stage embryo.

**Figure 9:**
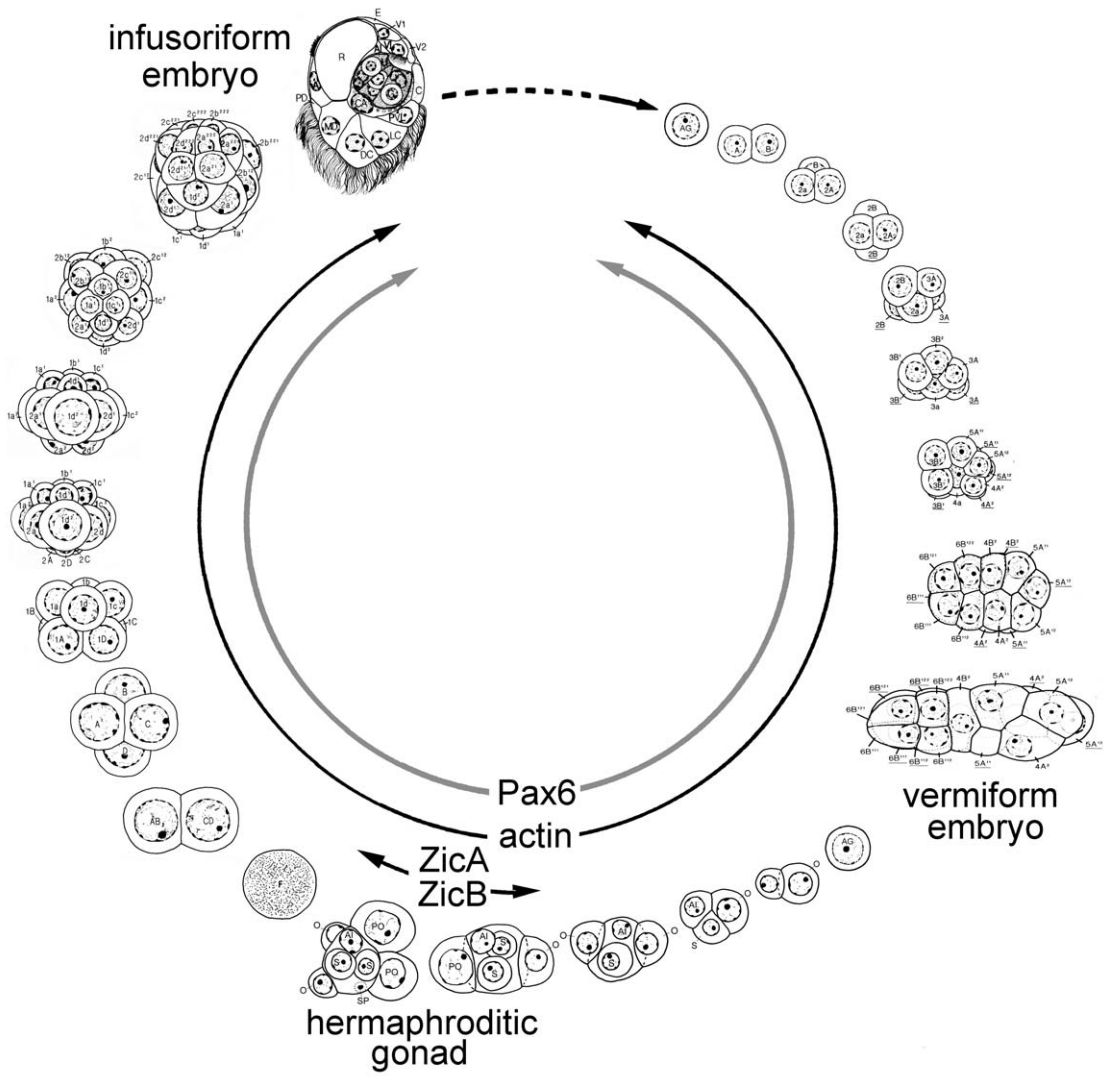
**Expression patterns of ZicA, ZicB, Pax6, and actin in developmental processes of *D. acuticephalum***. The development of the hermaphroditic gonad, vermiform embryo, and infusoriform embryo is based on Furuya et al. [50, 51, 52].

One of our main interests was the role of *Pax6 *and *Zic *in these highly simplified animals. To obtain clues, we examined the spatial expression profiles of these genes in *Dicyema *bodies. Dicyemid *ZicA *and *ZicB *were expressed in the hermaphroditic gonads (infusorigens) within the axial cells (Fig. 8A,D). No expression was detected in the embryonic stages (Fig. 8B,C). *Pax6 *was detected weakly throughout the developmental stages of the 2 types of embryo and in the hermaphroditic gonads, frequently only in the sperm (Fig. 8E). Thus, the hermaphroditic gonads expressed *Pax6*, *ZicA*, and *ZicB *most clearly (Fig. 9).

In contrast to the highly restricted expression of *Zic *genes, *actin1 *was expressed widely: in the agamete, hermaphroditic gonad, and the 2 types of developing embryo (Fig. 8F,G). *actin2 *showed an expression pattern very similar to that of *actin1 *(data not shown). In the hermaphroditic gonads (Fig. 8F), *actin1 *expression was detected in the sperm and the early-stage primary oocytes. In the developing infusoriform embryos, *actin1 *transcript was detected in blastomeres in the vegetal hemisphere of the embryo from the early to middle stages of development (Fig. 8F). In the late developmental stages, expression was restricted to the urn cells that consisted of the internal mass of the embryo (Fig. 8F). In the vermiform embryos, *actin1 *was expressed in the presumptive peripheral cells and agametes within the axial cell of the embryo (Fig. 8G).

## Discussion

### Phylogenetic position of dicyemids

Recent phylogenetic analysis of Pax genes revealed five main classes of Pax genes (*Pax4/6*, *Pax2/5/8/B*, *PaxA/C/Pox-neuro*, *Pax1/9*, and *Pax3/7/D*) [12,20]. In a comprehensive analysis [20], *Pax6 *and *Pax4 *homologues are limited to bilaterians and mammalians, respectively, even though a cnidarian genome project is completing [24]. Based on these backgrounds, we think that the grouping of the *Dicyema Pax *homologue with bilaterian *Pax6 *genes in this study supports the bilaterian origin of *Dicyema*.

In addition to the *Pax6 *phylogeny, the presence of introns at bilaterian-specific positions in the *ZicA *and *ZicB *ZF-encoding domains also favors the idea that dicyemids have bilaterian ancestry. Taken together with the results of previous molecular phylogenetic studies (*18S rDNA *[3], *Antennapedia *orthologue [4]), we can conclude that dicyemids are members of the Bilateria, and that dicyemids are simplified bilaterian animals. In fact, the infusoriform larvae of dicyemids have a bilateral body plan (Fig. 1). This feature seems to represent the true level of organization in dicyemids, because these larvae are free-swimming, whereas the adult stages are restricted to the renal organs of host cephalopods [25]. The bodies of the adult stages might have been simplified as a reflection of their specialization to their parasitic habitat – the tubular spaces of cephalopod renal organs [26].

Although we tried to delineate in more detail the origin of *Dicyema *by using other *Dicyema *sequences, including concatenated ones, the attempt was unsuccessful (data not shown). The difficulty seems to lie mainly in the highly diverged nature of the *Dicyema *sequences. Molecular phylogenetic analyses using other sets of slowly evolving sequences may improve our understanding.

### Accelerated molecular evolution of dicyemids

The five *Dicyema *sequences obtained in this study generally showed higher aa sequence diversification rates than the mean rates of other bilaterian orthologs. In a recent study, Thomas et al. [27] revealed that marked rate variations exist among the Echinodermata, Arthropoda, Mollusca, Annelida, and Platyhelminthes. They suggested that the existence of a strict molecular clock cannot be assumed for the Metazoa. The fact that we found general acceleration of the divergence rate in *Dicyema *may support this idea.

There are two circumstances under which lineage-specific differences in divergence rates may arise [27]: (1) alteration in the mutation rate of the species (e.g. a higher metabolic rate and shorter generation time may result in more mutations); and (2) differences in the proportion of mutations that become fixed in a population (e.g., species with smaller population sizes are expected to have faster rates of molecular evolution). In the case of dicyemids, both remain plausible, because only self-fertilization occurs in dicyemids [28,29]. However, we can at least speculate that the presence of an asexual reproductive cycle may accelerate the accumulation of mutations, because a recent study showed that transition to asexuality leads to an excess number of aa substitutions in a microcrustacean, *Daphnia pulex *[30]. Other possibilities, including shorter generation time and higher metabolic rate, remain open.

### Short and similarly-sized introns in dicyemids

Another critical finding about the *Dicyema *genome was the consistently short length of the introns in dicyemid genes, especially compared with other eumetazoans. Size distribution analyses have revealed that the mean lengths of human, *Ciona*, and *Drosophila *introns peak at 87, 60, and 57 bp, respectively [31]. *Dicyema *introns were even smaller than the average introns of fungi (*Schizosaccharomyces pombe*, 93 bp; *Aspergillus*, 72 bp) [32]. A recent study showed that the ciliated protozoan *Paramecium tetraurelia *has 20- to 35-bp introns (average size, 24.8 bp; n = 1061) [33]. This is very close to our mean intron size for *Dicyema *(26.2 bp). Although the intron size varies in the eukaryotes, *Dicyema *may belong to the lowest group.

There is currently no definitive answer to the biological significance of the smallness and uniformity of these intron sizes. However, we noticed that more extreme cases of intron size reduction appear in the nucleomorph of the alga *Bigelowella natans *[34]. The nucleomorph is a vestige of the eukaryotic cell nucleus after an endosymbiotic process in which one eukaryotic cell is engulfed by another. The sizes of the 852 introns in the *Bigelowella natans *nucleomorph genome are limited to either 18, 19, 20, or 21 bp. Considering the analogous situation between endosymbiosis and parasitism, the shortening of the introns in dicyemids may reflect a high level of selective pressure to reduce unnecessary expense (such as long introns) during the course of adaptive simplification. The size reduction may be facilitated by a lack of the need for the presumptive cis-regulatory elements essential for adapting to various environments.

### Gene-to-gene variation of the divergence profile across metazoan phyla

The *actin *sequences showed particularly strong diversification between *Dicyema *and other eumetazoa. This finding surprised us, because *actin *genes are widely conserved in eukaryotes, including in fungi and plants, in which *Pax6 *or *Zic *homologs have not yet been identified. *Dicyema actin *was expressed in the agamete, hermaphroditic gonad, and the developmental stages of the 2 types of embryo. Actin bundles play a role in many cellular processes, such as cell division. This suggests that more transcripts of *actin *may be required for cell division and cleavage during embryogenesis than in the external structure of the worms (calotte and peripheral cells). The actin bundles also contribute to cell movement; actin expression was therefore detected in unflagellated sperm in the dicyemids. Considering the essential role of actin in determining cell shape in multicellular animals, it is tempting to speculate that a reduction in somatic cell number, as is seen in *Dicyema*, may reduce the evolutionary constraint on actin protein structure.

### Pax6 and Zic genes in the highly simplified metazoa

Our major interest was in the features of *Dicyema Pax6 *and *Zic*, which are utilized in the developmental processes of many bilaterians. In this context, they can be regarded as "toolkit genes [35]," like the genes encoding Hox, parahox, and other developmentally critical transcription factors. Our most important finding may be the presence of both genes in the dicyemid. These genes could have been lost in the course of evolution. In particular, *Pax6 *is widely involved in photosensing organ development in bilaterians [5]. However, *Pax6 *PD/HD and *Zic *ZF were fairly well conserved, and their diversification rates were comparable to those of *ATPS *and *aldolase *and lower than that of *actin*.

Based on their expression profiles, the products of *ZicA *and *ZicB *may have a similar role in the hermaphroditic gonad within the axial cell. *Dicyema Zic *genes may therefore be associated with gametogenesis or the differentiation of gametes.

*Dicyema Pax6 *was detected throughout the developmental stages of the 2 types of embryo and in the hermaphroditic gonads. The location of this expression suggests that *Dicyema Pax6 *has few specific roles, such as the genetic control of eye development and neurogenesis. In this regard, previous studies showed various roles *Pax6 *in eumetazoan development. In *Caenorhabditis elegans*, its *Pax6 *homologue (*vab-3*) is required for the cell fate decision of a male specific blast cell lineage [36], and the control of hermaphrodite gonad size and shape [37]. In planarians, *Pax6A *is expressed not only in their nervous system but also in a non-cephalic parenchyma that gives rise to ventral marginal adhesive zone and is dispensable for eye regeneration [38]. Together with our results, the phylogenetic role of *Pax6 *may not be limited to the nervous system and photosensing organs.

The maintenance of the two toolkit genes in *Dicyema *that lack photosensing organs or nervous systems suggests that these genes possess unexpected versatility in the development of multicellular animals. The common expression of *Pax6 *and *Zic *in *Dicyema *hermaphroditic gonads suggests that they can be involved even in the most fundamental and indispensable function in multicellular animals, i.e. gametogenesis.

## Conclusion

The results in this study can be summarized as follows: 1) the *Pax6 *molecular phylogeny and *Zic *intron positions supported the idea of dicyemids as reduced bilaterians; 2) the aa sequences deduced from the 7 *Dicyema *genes were highly divergent in comparison to those from bilaterians; 3) dicyemid genes contained very short introns of uniform length; and 4) *ZicA *and *ZicB *were expressed in hermaphroditic gonads, and *Pax6 *was expressed weakly throughout the developmental stages of the 2 types of embryo and in the hermaphroditic gonads. The accelerated evolutionary rates and very short and uniform intron may represent a part of *Dicyema *genomic features. The presence and expression of the 2 tool-kit genes (*Pax6 *and *Zic*) in *Dicyema *suggests that they can be very versatile genes even required for the nervous-system-or-photosensing-organ-lacking bilaterian.

## Methods

### Animals

*Dicyema acuticephalum *was obtained from the renal organs of *Octopus vulgaris *purchased at a fish market in Osaka, Japan. The bodies of *D. acuticephalum*, which are 200–800 μm long, consist of a central cylindrical cell (the axial cell) and a single layer of 18 ciliated external cells (the peripheral cells; Fig. 1). In the anterior region, 8 peripheral cells form the calotte, which inserts the anterior region of the body into the crypts or folds in the renal organs of the host cephalopod. Species identity was confirmed by 18S rDNA phylogeny (AB266027, data not shown).

### Cloning and sequencing

Dicyemids were collected from the renal organs of the octopus with pipettes. Contaminating host tissues in the dicyemid suspension were removed with pipettes under a stereoscopic microscope. The isolated dicyemids were washed several times with artificial seawater.

Genomic DNA and total RNA were extracted as described [39]. For both nucleic acids, glycogen was used during precipitation to increase recovery. The cDNA fragments for *Pax6*, *Zic*, and *actin *were amplified by PCR using universal primers [15,40,41]. In a pilot molecular phylogeny analysis, it was clear that these genes were not orthologs of any other paired domain-containing genes (e.g. *Pax2*/*5*/*8*) or ZF motif-containing genes (e.g. *Gli *and *Glis*) (data not shown). On the basis of the sequences initially identified, 3' RACE (rapid amplification of cDNA-ends) PCR was done with a 3'-Full RACE Core Set (Takara BIO, Shiga, Japan). In the course of above PCR analyses, we obtained *ATP synthase beta subunit *and *fructose-bisphosphate aldolase *as PCR products of mispriming. Their nucleotide sequences were determined and deposited in DDBJ database. We considered that the cloned *ZicA*, *ZicB*, *Pax6*, and *actin1 *and *actin2 *cDNAs represented major molecular species in *D. acuticephalum*, because all of the PCR fragments (at least 15 for each gene) generated by universal primers contained sequences nearly identical to the first fragment we obtained. The 5' flanking genomic sequences were cloned with a DNA Walking SpeedUp Kit (Seegene, Seoul, Korea). Putative exons were identified by the open reading frame search program in DNASISPro (Hitachi Software Engineering, Tokyo, Japan) and by their similarity to homologs in other bilaterian species. Finally, entire coding regions were amplified from both cDNA and genomic DNA by using a pair of PCR primers that amplified the deduced open reading frames of the genes. The PCR products were cloned into pGEMT Easy Vector (Promega, Madison, WI). The nucleotide sequences were determined on the basis of the sequencing of at least 4 independent clones. 18S ribosomal DNA was cloned as described [15]. The sources of the cloned sequences were verified by highly stringent Southern blot analysis using genomic DNAs from both *D. acuticephalum *and the cephalopod *Octopus ocellatus *(data not shown).

### Molecular phylogenetic analysis

Deduced aa sequences were subjected to phylogenetic analysis. The other sequences were collected from NCBI database. The lists of the sequences with their species identity and accession number are indicated in Tables (see   Additional file 2).  . Sequences were aligned by using CLUSTALW [42] implemented in MEGA3 [43]. Regions with insertions or deletions resulting from the program were omitted by visual inspection. BI analysis was done with MrBayes 3.1.2 [44,45]. In BI analysis, we used an empirical model (JTT matrix, [46]) with Inv+gamma, alpha shape parameters, and aa frequencies estimated from the data. We ran 1,000,000 generations with 1 cold and 3 incrementally heated Markov chains, random starting trees for each chain, and sampling of trees every 100 generations. 50% major-rule consensus trees were constructed from the samples of stabilized trees. NJ and MP analyses were done with MEGA3.1 [43]. NJ tree was based on the distance calculation with JTT matrix [46] after removing position containing gaps (complete deletion option) for genes other than Zic genes, or after removing only in pairwise sequence comparisons (Pairwise deletion option) for Zic genes. For the NJ trees and MP tree analyses, the statistical significance of the branches obtained was calculated according to a bootstrap test [47] with 1000 repetitions. The alternative tree analysis was done as described [48]. Briefly, alternative trees were constructed by RETREE program, and the log-likelihood values were calculated by PROML program, taking into account site-specific rate differences using a gamma correction. Both programs are implemented in PHYLIP program package [49]. The statistical significance was determined by Shimodaira-Hasegawa test [21,22] implemented in PHYLIP.

For the aa substitution rate analysis, ancestral aa sequences for each protein were deduced by the ANCESCON program, a distance-based program that gives more accurate ancestral sequence reconstruction than do PAML, PHYLIP, and PAUP* at large evolutionary distances [23]with all of the bilaterian sequences. We used MEGA3.1 to calculate the pairwise distance. For the test of heterogeneity of variance (F-test), the aa substitution frequencies were first normalized by [(total aa numbers in the sequence) × (1-Pinv)].

### Whole-mount in-situ hybridization analysis of dicyemids

Parts of the cDNA fragments were ligated into pBluescript II KS vector, and we used this plasmid to synthesize a Dig (digoxygenin)-labeled RNA probe. Dig-labeled RNA probes were made by in vitro transcription from the linearized plasmid with a Dig RNA labeling kit (Roche). T7 or T3 RNA polymerase (Roche) was used in the in vitro transcription to synthesize an antisense RNA probe for hybridization.

In each step, it took 15 min to form a drop of dicyemids in the bottom of each microtube. Dicyemids were washed 5 times and then fixed overnight at 4°C in 4% paraformaldehyde in 0.5 M NaCl and 0.1 M MOPS buffer. After fixation, the dicyemids were dehydrated in an ethanol series (30%, 50%, 70%) for 10 min each and the dicyemids were stored in a 70% ethanol up-series at -30°C. Dicyemids were hydrated in an ethanol down-series (70%, 50%, 30%) for 5 min each, and were washed 3 times in PBT (phosphate buffer saline [PBS] with 0.1% Tween 20). The hydrated dicyemids were partly digested by protease K (0.5 mg/mL in PBS) for 15 min at 37°C.

After digestion, the dicyemids were washed in PBT and then fixed in 4% para- formaldehyde in PBS for 60 min at room temperature. After fixation, the dicyemids were washed 4 times in PBT for 5 min at room temperature. After washing, they were incubated in the following serial steps: in 50% hybridization buffer (5 × SSC, 1% SDS, 50% formamide) in PBT for 10 min at room temperature; in hybridization buffer for 10 min at room temperature; and in hybridization buffer for 1 h at 50°C. After incubation, the Dig-labeled RNA probe was added to the hybridization buffer and the mixture incubated overnight at 50°C.

The dicyemids were washed in the following serial steps: in 5 × SSC – 50% formamide – 1% SDS for 20 min at 50°C; in 2 × SSC – 50% formamide – 1% SDS for 20 min at 50°C; in 2 × SSCT (2 × SSC and 0.1%Tween) for 15 min at 50°C twice; in 1 × SSCT (1 × SSC and 0.1% Tween) for 15 min at 50°C twice; in 0.5 × SSCT (0.5 × SSC and 0.1% Tween) for 15 min at 50°C twice; and in 0.2 × SSCT (0.2 × SSC and 0.1% Tween) for 15 min at 50°C twice. After these washes, the dicyemids were washed for 5 min in a blocking buffer containing 2% BM blocking reagent (Roche); they were incubated in the blocking buffer for 60 min and then placed at 4°C for 10 min before the addition of antibody. An alkaline-phosphatase-conjugated anti-Dig antibody (Roche) was added at 1/2000 volume of the suspension of dicyemids. The antibody reaction was conducted overnight at 4°C. The dicyemids were then washed in PBT for 15 min at room temperature 4 times. After this, they were washed twice in a detection buffer (100 mM Tris-HCl [pH 9.5], 100 mM NaCl, 50 mM MgCl_2_) for 15 min at room temperature. After these washes, the dicyemids were immersed in BM purple solution (Roche) to detect the alkaline phosphatase activity.

We also conducted control experiments in the absence of probe or in the presence of a probe [DDBJ accession no. AB299857] that gave a spatially restricted signals. The specificity of the signals was checked with these two controls in each hybridization analysis.

## Abbreviations

aa, amino acid; Aldolase, fructose-bisphosphate aldolase; ATP synthase (ATPS), ATP synthase beta subunit; BI, Bayesian inference; HD, homeodomain; MP, maximal parsimony; NJ, neighbour-joining; PD, paired box domain; ZF, zinc finger domain.

## Authors' contributions

JA conceived of study, carried out the molecular cloning and the molecular phylogenetic analysis, and drafted the manuscript. YSO carried out in situ hybridization analysis. AK carried out the molecular cloning. HF participated in the design of the study, carried out in situ hybridization analysis, and drafted the manuscript. All authors read and approved the final manuscript.

## Supplementary Material

Additional file 1 Supplemental phylogenetic trees. Supplemental Fig. 1 – NJ tree of Pax6. Supplemental Fig. 2 – MP tree of Pax6. Supplemental Fig. 3 – NJ tree of Actin. Supplemental Fig. 4 – NJ tree of ATP synthase. Supplemental Fig. 5 – NJ tree of Aldolase. Click here for file

Additional file 2 List of sequences used in this study. Supplemental Table 1 – Pax sequences. Supplemental Table 2 – Zic sequences. Supplemental Table 3 – Actin sequences. Supplemental Table 4 – ATP synthase beta subunit sequences. Supplemental Table 5 – Aldolase sequences. Click here for file
