# Supplementary material for: Dicyema Pax6 and Zic: tool-kit genes in a highly simplified bilaterian
Source: BMC Evol Biol. 2007 Oct 25;7:201. doi: 10.1186/1471-2148-7-201 (PMC2222250; doi:10.1186/1471-2148-7-201)
Supplement: Additional file 1 — Supplemental phylogenetic trees. Supplemental Fig. 1 – NJ tree of Pax6. Supplemental Fig. 2 – MP tree of Pax6. Supplemental Fig. 3 – NJ tree of Actin. Supplemental Fig. 4 – NJ tree of ATP synthase. Supplemental Fig. 5 – NJ tree of Aldolase. [file 1471-2148-7-201-S1.pdf]

Supplemental figures for Aruga et al., “Dicyema Pax6 and Zic : tool-kit genes in a highly simplified bilaterian”

Figure legends for Supplemental Figures

Supplemental Fig. 1

NJ tree of Pax6 proteins

The trees were drawn by MEGA3 by using PD (A) or concatenated PD+HD (B) sequences. PD tree was unrooted. PD+HD tree was rooted with PaxB family of which subtree is indicated in closed triangle. Numerals on interior branches indicate bootstrap values greater than 50%. The scale bar represents evolutionary distance in substitutions/aa residue. Classification of Pax genes is indicated in (A) according to Matus et al. (2007).

Supplemental Fig. 2

Unrooted MP tree of Pax6 proteins

MP tree was drawn by MEGA3 using concatenated PD+HD sequences. Numerals on interior branches indicate bootstrap values in percentages. Interior branches with lower than 70% bootstrap values were condensed. 10 out of 10 most parsimonious trees shows the same condensed tree pattern. There were a total of 194 positions, out of which 86 were parsimony informative.

Supplemental Fig. 3

Unrooted NJ tree of Actin proteins

The sequences of 63 metazoan Actin sequences underwent multiple alignment and tree

analysis. Gray circles indicate taxonomic groups (species names are removed after constructing tree). Taxonomic information on the sequences used is given in Supplemental Table 3. Species names are indicated only for *Dicyema* and animals belonging to Platyhelminthes (*Echinococcus granulosus* [class Cestoda], *Diphyllobothrium dendriticum* [class Cestoda], *Schistosoma mansoni* [class Trematoda], *Schistosoma japonicum* [class Trematoda], *Girardia tigrina* [class Turbellaria]). Numerals on interior branches indicate bootstrap value in percentages of any branches that showed more than 90% in bootstrap test. The scale bar represents evolutionary distance in substitutions/amino acid residue.

Supplemental Fig. 4

Unrooted NJ tree of ATP synthase beta subunit proteins. Numerals on interior branches indicate bootstrap values more than 90%. The scale bar represents evolutionary distance in substitutions/amino acid residue.

Supplemental Fig. 5

Unrooted NJ tree of fructose-bisphosphate aldolase proteins. Numerals on interior branches indicate bootstrap values more than 90%. The scale bar represents evolutionary distance in substitutions/amino acid residue. Gray circles indicate taxonomic groups.

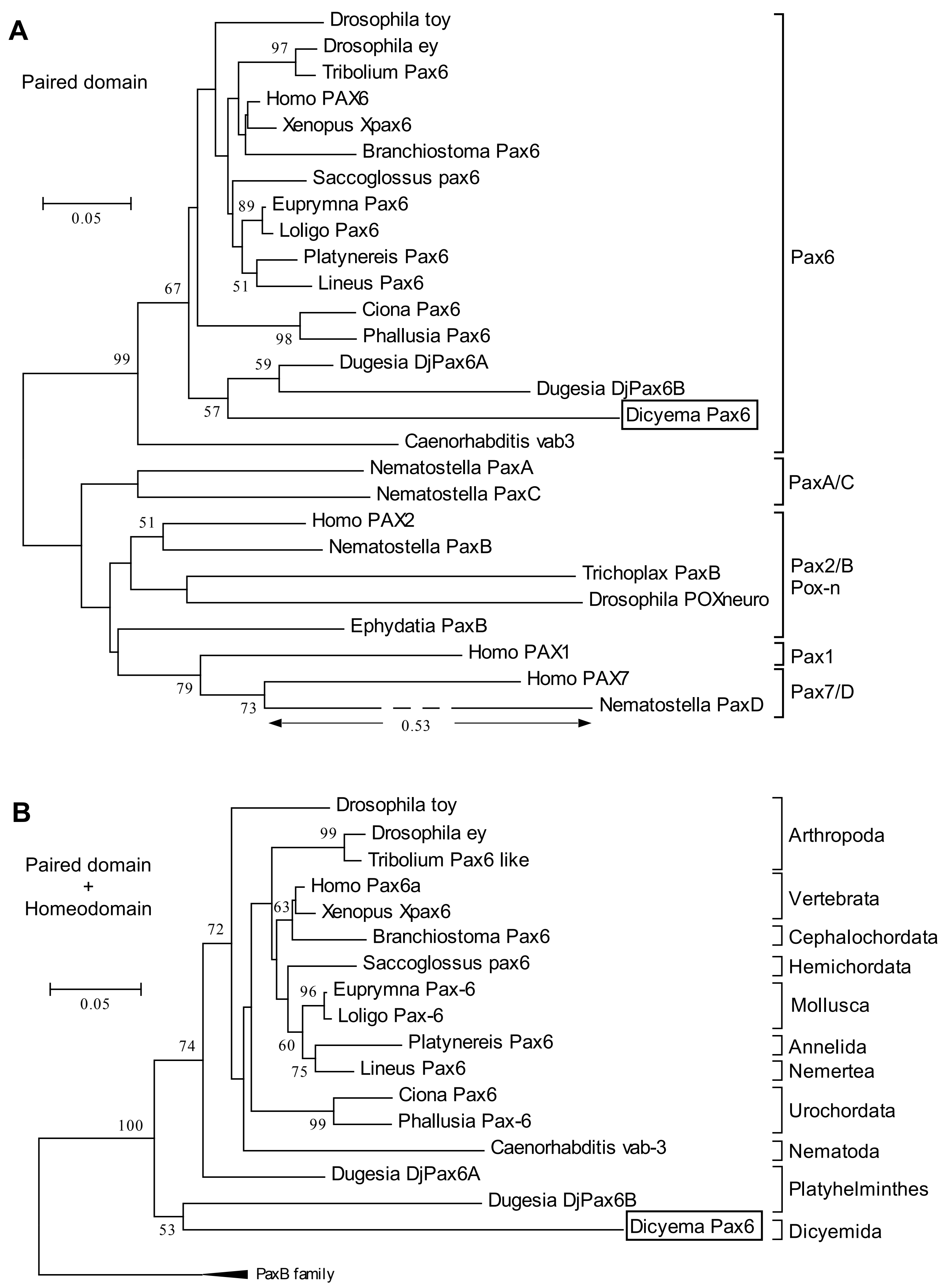

Supplemental Fig. 1

Pax6 NJ tree

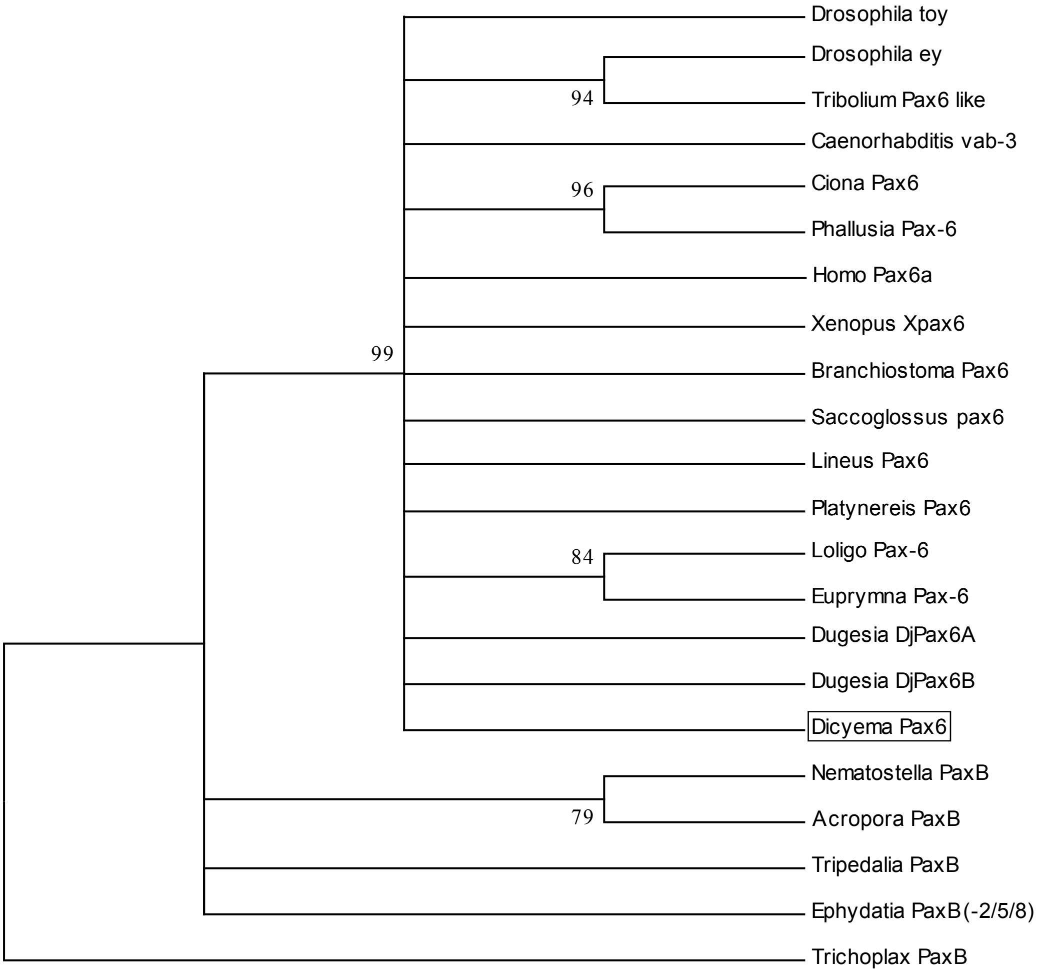

Supplemental Fig. 2

Pax6 PD+HD MP tree

(70% major-rule condensed tree)

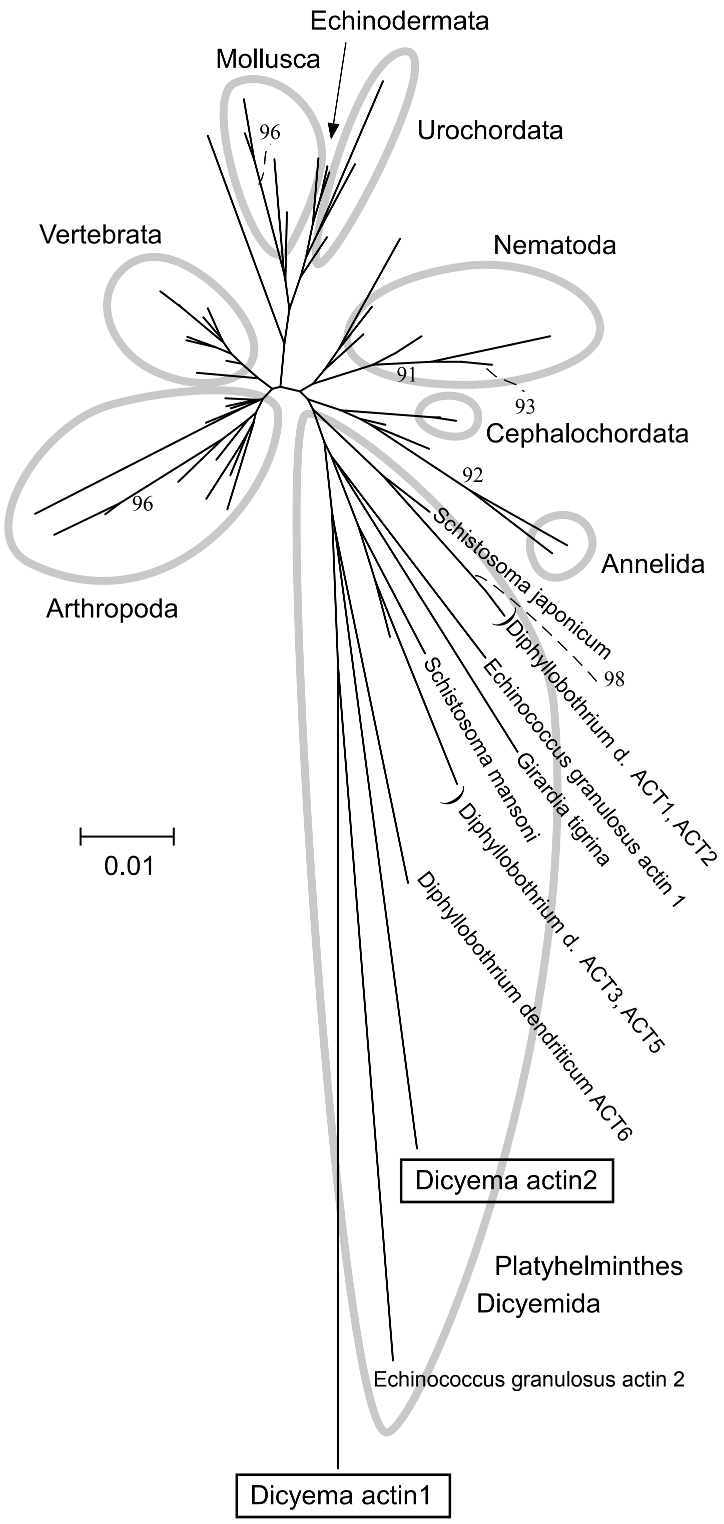

Supplemental Fig. 3

Actin NJ tree

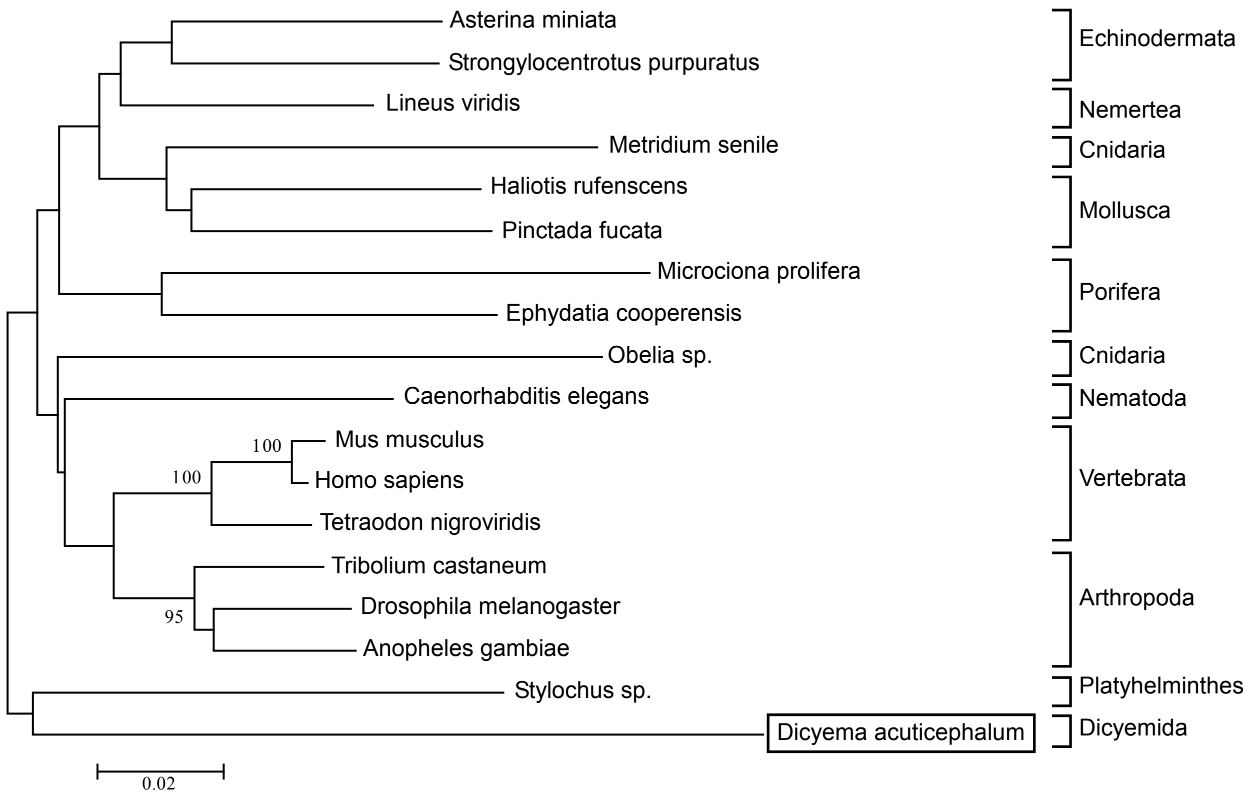

Supplemental Fig. 4

ATP synthase beta subunit NJ tree

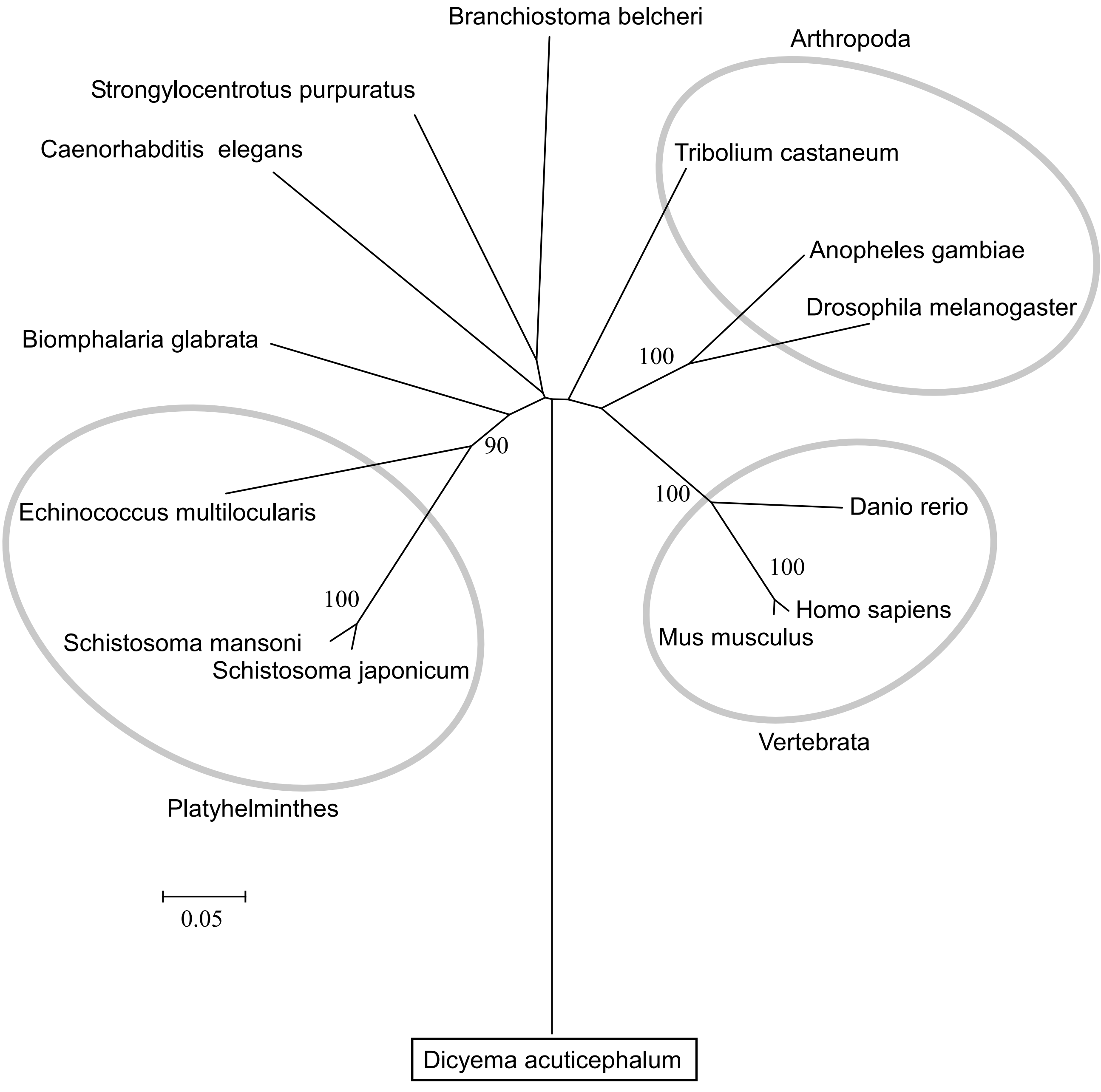

Supplemental Fig. 5

fructose-bisphosphate aldolase NJ tree
