# Supplementary material for: Dicyema Pax6 and Zic: tool-kit genes in a highly simplified bilaterian
Source: BMC Evol Biol. 2007 Oct 25;7:201. doi: 10.1186/1471-2148-7-201 (PMC2222250; doi:10.1186/1471-2148-7-201)
Supplement: Additional file 2 — List of sequences used in this study. Supplemental Table 1 – Pax sequences. Supplemental Table 2 – Zic sequences. Supplemental Table 3 – Actin sequences. Supplemental Table 4 – ATP synthase beta subunit sequences. Supplemental Table 5 – Aldolase sequences. [file 1471-2148-7-201-S2.pdf]

Supplemental Table 1 Pax sequences

Phylum	Animal species	Gene name	Genome sequence	cDNA or aa sequence
Dicyemida	*Dicyema acuticephalum*	Pax6	AB266034	AB266035
Annelida	*Platynereis dumerilii*	Pax6		CAJ40659
Arthropoda	*Drosophila melanogaster*	toy		NP_524638
Arthropoda	*Drosophila melanogaster*	ey *		O18381
Arthropoda	*Drosophila melanogaster*	Pox-neuro		P23758
Arthropoda	*Tribolium castaneum*	Pax6 like		XP_975543
Cephalochordata	*Branchiostoma floridae*	Pax6		CAA11366
Cnidaria	*Tripedalia cystophora*	PaxB		AAQ17211
Cnidaria	*Acropora millepora*	PaxB		AAF64460
Cnidaria	*Nematostella vectensis*	PaxA		AAW29066
Cnidaria	*Nematostella vectensis*	PaxB		AAW29067
Cnidaria	*Nematostella vectensis*	PaxC		AAW29068
Cnidaria	*Nematostella vectensis*	PaxD		AAW29069
Hemichordata	*Saccoglossus kowalevskii*	pax6		AAP79294
Mollusca	*Loligo opalescens*	Pax-6		AAB40616
Mollusca	*Eupryma scolopes*	Pax6		AAM74161
Nematoda	*Caenorhabditis elegans*	vab-3		NP_001024572
Nemertea	*Lineus sanguineus*	Pax6-like		CAA64847
Placozoa	*Trichoplax adhaerens*	PaxB		AAY68376
Platyhelminthes	*Dugesia japonica*	Dj-Pax6(A)		BAA75672
Platyhelminthes	*Dugesia japonica*	Dj-Pax6B *		CAC85262
Porifera	*Ephydatia fluviatilis*	Pax-2/5/8		BAA36346
Urochordata	*Ciona intestinalis*	Pax6		NP_001027641
Urochordata	*Phallusia mammilata*	Pax-6		CAA71094
Vertebrata	*Xenopus leavis*	MGC52531		AAH41712
Vertebrata	*Homo sapiens*	PAX1		NP_006183
Vertebrata	*Homo sapiens*	PAX2		NP_003978
Vertebrata	*Homo sapiens*	PAX6		NP_000271
Vertebrata	*Homo sapiens*	PAX7		NP_002575

*, sequences not used in the aa substitution rate analysis.

Supplemental Table 2 Zic sequences

Phylum	Animal species	Gene name	Genome sequence	cDNA or aa sequence
Dicyemida	*Dicyema acuticephalum*	ZicA	AB266036	AB266039
Dicyemida	*Dicyema acuticephalum*	ZicB	AB266037	AB266038
Annelida	*Tubifex tubifex*	Tt-Zic	AB231869	AB231870
Arthropoda	*Drosophila melanogaster*	Opa		NP_524228
Arthropoda	*Anopheles gambiae*	ENSANG00000009802		XM_321856
Arthropoda	*Pandinus imperator*	Pi-Zic	AB231876	AB231877
Arthropoda	*Artemia franciscana*	Af-Zic	AB231878	AB231879
Cephalochordata	*Branchiostoma floridae*	AmphiZic	AB231866	BAE94124
Cnidaria	*Scolionema suvaense*	Ssu-Zic	AB231882	AB231883
Cnidaria	*Hydra vulgaris*	HyZic		AAR10817
Cnidaria	*Nematostella vectensis*	Nv-ZicA	AB231867	
Cnidaria	*Nematostella vectensis*	Nv-ZicE	AB231868	
Echinodermata	*Strongylocentrotus purpuratus*	Sp-Zic	[ref. in 14]	
Echinodermata	*Asterina pectinifera*	Ap-Zic	AB231871	AB231872
Mollusca	*Loligo bleekeri*	Lb-Zic		AB231874
Mollusca	*Octopus ocellatus*	Oo-Zic		AB231875
Mollusca	*Corbicula* sp.	Cj-Zic	AB231873	
Mollusca	*Spisula solidissima*	Sso-Zic	AB231865	
Nematoda	*Caenorhabditis elegans*	Ref-2		AAM55473
Platyhelminthes	*Dugesia japonica*	Dj-ZicA		AB231880
Platyhelminthes	*Dugesia japonica*	Dj-ZicB		AB231881
Platyhelminthes	*Schistosoma mansoni*	Sma-Zic	AB231864	
Urochordata	*Ciona intestinalis*	ZicL		NP_001027958
Urochordata	*Ciona intestinalis*	macho1		BAE06762
Urochordata	*Halocynthia roretzi*	ZicN		BAC23063,
Urochordata	*Halocynthia roretzi*	macho1		BAB19958
Vertebrata	*Xenopus laevis.*	Zic2		AB009565
Vertebrata	*Xenopus laevis.*	Zic5		AB034983
Vertebrata	*Mus musculus*	Zic2 *		NP_033600
Vertebrata	*Mus musculus*	Zic5 *		NP_075363
Vertebrata	*Homo sapiens*	ZIC2		NP_009060

Vertebrata	*Homo sapiens*	ZIC5		NP_149123

*, sequences not used in the aa substitution rate analysis.

Supplemental Table 3　Actin sequences

Phylum	Animal species	Gene name	Genome sequence	cDNA or aa sequence
Dicyemida	*Dicyema acuticephalum*	actin1	AB266028	AB266029
Dicyemida	*Dicyema acuticephalum*	actin2	AB267841	AB267840
Annelida	*Helobdella triserialis*	actin		CAA04046
Annelida	*Lumbricus terrestris*	Actin		CAA65362
Arthropoda	*Aedes aegypti*	actin		AAA62350
Arthropoda	*Artemia franciscana*	actin		ABG27014
Arthropoda	*Apriona germari*	actin		AAV65298
Arthropoda	*Apis mellifera*	actin		XP_623826
Arthropoda	*Daphnia magna*	actin		CAB99474
Arthropoda	*Drosophila melanogaster*	actin		BAA20058
Arthropoda	*Helicoverpa armigera*	actA3a		CAA66218
Arthropoda	*Homarus gammarus*	beta actin		CAE46725
Arthropoda	*Haemaphysalis longicornis*	actin		AAP81255
Arthropoda	*Ixodes ricinus*	actin		CAI63975
Arthropoda	*Lymantria dispar*	actin		AAD54427
Arthropoda	*Limulus polyphemus*	actin		CAA86289
Arthropoda	*Ornithodoros moubata*	actin		BAE46505
Arthropoda	*Rhipicephalus appendiculatus*	actin		AAP81256
Arthropoda	*Toxoptera citricida*	putative actin		AAU84923
Cephalochordata	*Branchiostoma belcheri*	Actin cytoplasmic		BAA13444
Cephalochordata	*Branchiostoma floridae*	Actin cytoplasmic		BAA13350
Echinodermata	*Strongylocentrotus purpuratus*	actin		NP_999634
Echinodermata	*Tripneustes gratilla*	CyI actin		AAB31965
Hemichordata	*Saccoglossus kowalevskii*	actin		CAA74016
Mollusca	*Biomphalaria tenagophila*	actin		AAK68714
Mollusca	*Chlamys farreri*	actin		AAP88387
Mollusca	*Crassostrea gigas*	Actin 2		BAB84579
Mollusca	*Dreissena polymorpha*	cytoplasmic actin		AAC32224
Mollusca	*Haliotis iris*	actin A1		AAX19286
Mollusca	*Helisoma trivolvis*	actin		AAK68715
Mollusca	*Loligo pealei*	beta actin		AAU11523

Nematoda	*Caenorhabditis elegans*	actin		CAA34718
Nematoda	*Cooperia oncophora*	actin		AAR21857
Nematoda	*Globodera rostochiensis*	actin		AAN15196
Nematoda	*Onchocerca volvulus*	Actin-1A		A48449
Nematoda	*Plectus acuminatus*	actin		CAA10111
Nematoda	*Panagrellus redivivus*	actin		AAQ89578
Nematoda	*Setaria digitata*	actin		AAD13153
Nematoda	*Wuchereria bancrofti*	actin		AAF25819
Platyhelminthes	*Diphyllobothrium dendriticum*	Actin-1/4		P68556
Platyhelminthes	*Diphyllobothrium dendriticum*	Actin-2 *		P53456
Platyhelminthes	*Diphyllobothrium dendriticum*	Actin-3 *		P53457
Platyhelminthes	*Diphyllobothrium dendriticum*	Actin-5 *		P53458
Platyhelminthes	*Diphyllobothrium dendriticum*	Actin-6 *		P53459
Platyhelminthes	*Echinococcus granulosus*	actin 1		AAC37175
Platyhelminthes	*Echinococcus granulosus*	actin 2 *		AAC80574
Platyhelminthes	*Girardia tigrina*	actin		AAD11530
Platyhelminthes	*Schistosoma japonicum*	actin		AAW27501
Platyhelminthes	*Schistosoma mansoni*	Actin-1		P53470
Urochordata	*Ciona Savignyi*	CsCa1		BAA25398
Urochordata	*Halocynthia roretzi*	HrCA1		BAA08112
Urochordata	*Molgula occulta*	cytoskeletal actin 1		AAC28357
Urochordata	*Oikopleura longicauda*	cytoplasmic actin		BAA86216
Vertebrata	*Acanthopagrus schlegelii*	beta actin		AAR84618
Vertebrata	*Danio rerio*	Actin		NP_001032193
Vertebrata	*Epinephelus coioides*	beta actin		AAR97600
Vertebrata	*Physalaemus pustulosus*	beta actin		AAO67718
Vertebrata	*Rivulus marmoratus*	beta actin 2		AAV97945
Vertebrata	*Lethenteron japonicum*	cytoplasmic actin		BAB41207
Vertebrata	*Monopterus albus*	beta-actin		AAT69683
Vertebrata	*Oryctolagus cuniculus*	gamma non-muscle actin		CAA43140
Vertebrata	*Oncorhynchus mykiss*	beta-actin		AAF80342
Vertebrata	*Pagrus major*	beta cytoplasmic actin		BAD88412
Vertebrata	*Sigmodon hispidus*	beta actin		AAL16942
Vertebrata	*Triakis scyllium*	beta actin		BAB91355

*, sequences not used in the aa substitution rate analysis.

Supplemental Table 4　ATP synthase beta subunit sequences

Phylum	Animal species	Gene name	Genome sequence	cDNA or aa sequence
Dicyemida	*Dicyema acuticephalum*	ATP synthase beta subunit	AB266032	AB266033
Arthropoda	*Anopheles gambiae*	ENSANGP00000016863		XP_320445
Arthropoda	*Drosophila melanogaster*	ATP synthase beta subunit		CAA50332
Arthropoda	*Tribolium castaneum*	similar to CG11154-PA, isoform A		XP_972481
Cnidaria	*Metridium senile*	ATP synthase beta subunit		AAT06144
Cnidaria	*Obelia* sp.	ATP synthase beta subunit		AAT06143
Echinodermata	*Asterina miniata*	ATP synthase beta subunit		AAT06134
Echinodermata	*Strongylocentrotus purpuratus*	similar to CG11154-PA, isoform A		XP_783293
Mollusca	*Haliotis rufenscens*	ATP synthase beta subunit		AAZ30686
Mollusca	*Pinctada fucata*	ATP synthase beta subunit		ABC86835
Nematoda	*Caenorhabditis elegans*	Atp synthase subunit protein 2		AAA19068
Nemertea	*Lineus viridis*	ATP synthase beta subunit		AAZ30652
Platyhelminthes	*Stylochus* sp.	ATP synthase beta subunit		AAT06145
Porifera	*Ephydatia cooperensis*	ATP synthase beta subunit		AAT06136
Porifera	*Microciona prolifera*	ATP synthase beta subunit		AAZ30673
Vertebrata	*Homo sapiens*	F1 beta subunit		BAA00016
Vertebrata	*Mus musculus*	ATP synthase, H+ transporting mitochondrial F1 complex, beta subunit		BAE30095
Vertebrata	*Tetraodon nigroviridis*	unnamed protein product		CAG04958

Supplemental Table 5　Aldolase sequences

Phylum	Animal species	Gene name	Genome sequence	cDNA or aa sequence
Dicyemida	*Dicyema acuticephalum*	fructose-bisphosphate aldolase	AB266030	AB266031
Arthropoda	*Tribolium castaneum*	similar to CG6058-PF, isoform F isoform 3		XP_975842
Arthropoda	*Anopheles gambiae*	ENSANGP00000012760		EAA08079
Arthropoda	*Drosophila melanogaster*	fructose-bisphosphate aldolase (EC 4.1.2.13) 4 alpha		JX0233
Cephalochordata	*Branchiostoma belcheri*	aldolase		BAA21101
Echinodermata	*Strongylocentrotus purpuratus*	similar to aldolase a, fructose-bisphosphate		XP_790001
Mollusca	*Biomphalaria glabrata*	fructose-biphosphate aldolase		AAZ39527
Nematoda	*Caenorhabditis elegans*	Fructose-bisphosphate aldolase 1		P54216
Platyhelminthes	*Schistosoma japonicum*	SJCHGC00411		AAW25258
Platyhelminthes	*Schistosoma mansoni*	fructose bisphosphate aldolase		AAB84014
Platyhelminthes	*Echinococcus multilocularis*	fructose-bisphosphate-aldolase		CAC18550
Vertebrata	*Danio rerio*	Aldolase a, fructose-bisphosphate, a		AAH44379
Vertebrata	*Homo sapiens*	ALDOA		CAG46678
Vertebrata	*Mus musculus*	Aldolase 1, A isoform		AAH43026
